# Planning cooler cities through integration of UAV thermal imagery and GIS in local climate zone studies

**DOI:** 10.1038/s41598-025-25178-y

**Published:** 2025-12-06

**Authors:** Gökçe Gönüllü Sütçüoğlu, Ayşe Kalaycı

**Affiliations:** 1 Department of Urban Regeneration, İzmir Katip Çelebi University, İzmir, Türkiye; 2 Department of City and Regional Planning, İzmir Katip Çelebi University, İzmir, Türkiye

**Keywords:** Local climate zones, Urban heat island, Urban climate, Unmanned aerial vehicle, Thermal imaging, Geographic information systems, Climate sciences, Ecology, Ecology, Engineering, Environmental sciences

## Abstract

Urban microclimates result from complex interactions between buildings, vegetation, and human activities, impacting energy consumption, air quality, and urban planning. Understanding and mapping these microclimates is essential for sustainable city development. Geographic Information Systems (GIS) play a crucial role in analyzing microclimate patterns by integrating spatial datasets such as land cover, building heights, and meteorological data. This study examines urban microclimates in İzmir’s Konak District using GIS and unmanned aerial vehicles (UAVs) equipped with thermal sensors. By classifying Local Climate Zones (LCZs) and analyzing their relationship with land surface temperatures (LSTs), the research highlights how urban morphology shapes microclimatic conditions. The study area was divided into 2,435 grids, with UAV-based thermal imaging providing high-resolution temperature data. Findings indicate that LCZs with high impermeable surface fractions (e.g., LCZ 7, LCZ 8, and LCZ E) exhibited elevated temperatures, while vegetated or water-rich zones (e.g., LCZ B and LCZ G) demonstrated cooling effects. The Heat Load Map identified 8.8% of the district as experiencing excessive heat, while 21.7% benefited from optimal thermal conditions due to green and blue spaces. This study underscores the importance of increasing vegetation and permeable surfaces to mitigate urban heat islands (UHIs). By integrating UAV technology with GIS, it advances LCZ-based urban climate research and provides practical tools for climate-responsive planning. Understanding microclimates in dense urban areas enables targeted strategies to reduce heat stress, improve air quality, and enhance urban livability.

## Introduction

Urban microclimates—resulting from the interactions among urban form, surface materials, vegetation, and anthropogenic heat emissions—significantly influence thermal comfort, energy demand, and health outcomes in cities, making them a central focus in climate-responsive planning^1–3^. The Urban Heat Island (UHI) effect, characterized by elevated temperatures in urban areas relative to their rural surroundings, has been shown to intensify in response to climate change, urban densification, and the degradation of vegetated surfaces^4,5^. These factors collectively exacerbate thermal disparities within cities and amplify public health and energy concerns during heatwave events. UHI increases energy consumption, degrades air quality, and heightens vulnerability to heat-related health issues, particularly during extreme heat events^6,7^.

Understanding UHI’s spatial variability within cities remains challenging due to both the limited resolution of conventional satellite data and the morphological complexity of urban landscapes^8^. In response to this challenge, urban climate researchers have increasingly turned to classification schemes that systematically account for morphological and functional characteristics. Among these, the Local Climate Zone (LCZ) framework introduced by Stewart and Oke^6^ has become the most widely adopted, as it provides a standardized typology for mapping urban and natural surfaces based on structure, land cover, and function. Its 17-zone classification (10 built types and 7 land cover types) enables global comparative studies and forms the basis of initiatives such as the World Urban Database and Access Portal Tools (WUDAPT), which aim to produce standardized LCZ maps for cities worldwide^9–11^.

LCZ classification has often been combined with satellite imagery, GIS, and machine learning to analyze surface UHI (SUHI) patterns. While this approach has yielded valuable insights, especially for large-scale inter-city comparisons, the coarse resolution of satellite-derived Land Surface Temperature (LST) data frequently limits its usefulness for fine-scale urban planning^12,13^. For instance, Chen et al.^14^ in Chenzhou and Geletič & Lehnert^15^ in Olomouc demonstrated how LCZ-based mapping can reveal intra-urban temperature variations; however, their reliance on satellite-based LST data underscores the challenge of capturing detailed urban morphology with sufficient accuracy. This limitation has motivated emerging research to explore unmanned aerial vehicles (UAVs) as a flexible, high-resolution alternative capable of overcoming the spatial constraints of satellite observations.

Recent studies have shown that UAVs equipped with thermal sensors can provide sub-meter scale resolution, enabling highly precise neighborhood-scale temperature monitoring^16–18^. For example, Kim et al.^17^ demonstrated that UAV-derived thermal data captured land cover temperature differences in Korean urban green spaces with greater accuracy than satellite-derived LST, while Webster et al.^19^ employed UAVs to characterize vegetation canopies thermally in three dimensions. Together, these studies highlight how UAV-based thermal remote sensing has rapidly advanced the analytical scope of UHI research.

Beyond offering higher spatial resolution, UAVs enable monitoring of UHI intensity across multiple heights and times of day, providing crucial data for designing both vertical and temporal interventions. This capacity supports adaptive strategies such as building design modifications and time-sensitive cooling measures, which are particularly relevant for enhancing climate resilience in medium-sized and rapidly growing cities^20,21^. Moreover, the fine-grained thermal information generated by UAVs facilitates the detection of micro-urban heat islands (MUHIs) and localized hotspots that are often invisible to satellite-based analyses. Such detailed identification strengthens the potential for targeted mitigation strategies, from optimizing vegetation placement to planning energy consumption more effectively in heterogeneous urban landscapes^22–24^.

Despite advancements in high-resolution data acquisition and LCZ classification, studies systematically integrating UAV-derived thermal imagery with LCZ frameworks remain limited, often emphasizing classification accuracy or satellite-based LST analyses without delving into intra-zone temperature variations^16^. Studies show the integration of UAV-derived thermal data with LCZ classification enhances the spatial resolution and contextual relevance of urban heat assessments, enabling precise SUHI intensity mapping and temporal monitoring during heatwaves. This integration supports urban planning by providing actionable data for heat mitigation, energy efficiency measures, and climate adaptation strategies, as demonstrated in various case studies across different cities^21,25,26^. UAV-based LCZ analysis facilitates the development of localized urban heat hazard models and informs sustainable urban development decisions.

UAV and LCZ integrated approaches inform urban heat mitigation strategies by identifying high-risk zones and evaluating cooling effects of urban green spaces, albedo modifications, and morphological adjustments. These data support policy and design decisions aimed at improving outdoor thermal comfort, reducing energy consumption, and enhancing urban resilience to climate change^21,26–29^. Findings underscore the practical utility of fine-scale thermal and morphological data for sustainable urban development.

Deep learning models, object detection algorithms, and machine learning-based clustering techniques are increasingly applied to enhance LCZ classification accuracy and to model UHI dynamics effectively. These methods improve the semantic understanding of urban landscapes from high-resolution imagery and enable the identification of critical variables affecting UHI, such as building density and vegetation extent. The incorporation of AI-driven approaches facilitates automated, scalable analyses supporting urban climate resilience studies^26,30,31^.

Few studies have systematically combined UAV-derived thermal data with LCZ classification to assess surface temperature variations across entire urban districts. Where UAV studies exist, they typically focus on isolated features such as parks^17^ or infrastructure^32^, leaving a gap in large-scale applications that integrate both built and natural LCZ classes. Furthermore, Mediterranean cities, which are highly vulnerable to heat stress due to their dense urban forms and climatic conditions, remain underrepresented in UAV-based LCZ research.

The potential of UAV imagery to capture dynamic microclimatic variations and inform adaptive planning remains underutilized. In response to this research gap, this study proposes a localized, integrative approach. This study addresses this gap by integrating UAV-based LST measurements with GIS-derived LCZ classifications in Konak, İzmir, a dense urban area with diverse building morphologies and land uses. Combining thermal imagery from UAV flights with municipal zoning and functional data, the methodology classifies LCZs, assigns statistical temperature profiles, identifies high-heat-load zones, and simulates thermal impacts of future planning scenarios.

The novelty of this study lies in its empirical integration of UAV-derived thermal imagery with LCZ classification and spatial statistics to assess intra-zone temperature variability—an approach rarely applied at the neighborhood scale in high-density Mediterranean urban contexts. While satellite-based approaches offer broader coverage, they often lack the spatial resolution necessary to capture intra-urban heterogeneity. UAVs, by contrast, enable high-resolution temperature measurements but have rarely been applied systematically to classify LCZs and generate heat load maps for dense metropolitan areas. By combining real-time thermal mapping with zoning and functional land use data within a GIS framework, the study develops a replicable methodology that enables planners to identify high-risk heat exposure zones and simulate planning interventions. These contributions support climate-sensitive urban design and align with global policy frameworks such as the UN Sustainable Development Goals (Goal 11) and the New Urban Agenda, both of which emphasize data-driven resilience strategies for cities.

## Materials and methods

### Study area

**Fig. 1:**
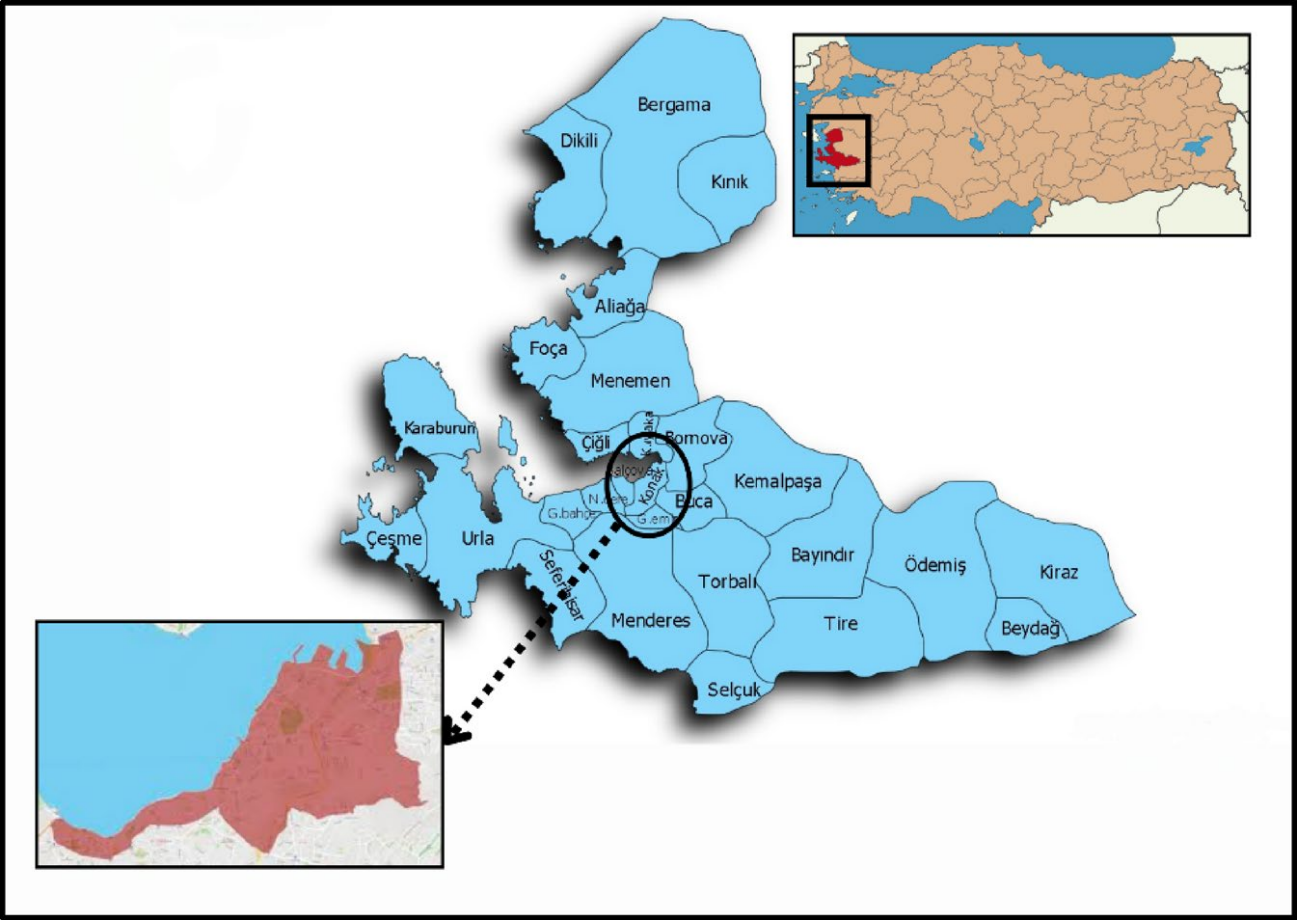
Study area (created by combining images adapted from^33–35^.

The study was conducted in Konak District, located at the core of İzmir, Turkey (Fig. 1). Konak serves as the historical, administrative, and commercial center of the city, hosting landmarks such as the Kemeraltı Bazaar, Konak Square, and the İzmir Clock Tower. While these cultural and historical features highlight the district’s importance, the urban morphology and environmental conditions are of particular relevance for climate and UHI studies. Konak is characterized by dense and heterogeneous land use, including commercial areas, residential neighborhoods, industrial zones, and port facilities. Its compact street network and mix of traditional and modern buildings contribute to diverse microclimatic conditions.

Climatically, Konak falls within the Mediterranean climate zone, with hot, dry summers and mild, wet winters. The annual average temperature is approximately 18–19 °C, with mean monthly temperatures ranging from around 10 °C in January to 28–30 °C in July and August^36^. Prevailing winds are dominated by northwesterly sea breezes (locally known as *İmbat*) during the summer, which provide natural cooling, while southerly winds are more common in winter months. Average annual precipitation is about 700–800 mm, concentrated between November and March.

The built environment of Konak predominantly features reinforced concrete structures with flat or tiled roofs, asphalt-paved roads, and extensive impervious surfaces. In the historic bazaar, narrow streets with stone pavements and metal or tile-roofed buildings are common, while modern residential and commercial zones employ concrete and glass as primary materials. Vegetation cover is sparse in the central areas, limited mostly to small urban parks, scattered street trees, and coastal green corridors. Typical plant species include Mediterranean evergreens such as olive (*Olea europaea*) and laurel (*Laurus nobilis*), as well as ornamental trees like plane (*Platanus orientalis*) and pine (*Pinus pinea*).

This combination of high-density built-up areas, limited vegetation, and climatic conditions makes Konak a suitable case study for exploring intra-urban thermal variability. The district’s heterogeneous urban morphology aligns well with the Local Climate Zone (LCZ) framework, enabling systematic classification and detailed examination of UHI effects at the neighborhood scale.

### Methodology

**Fig. 2:**
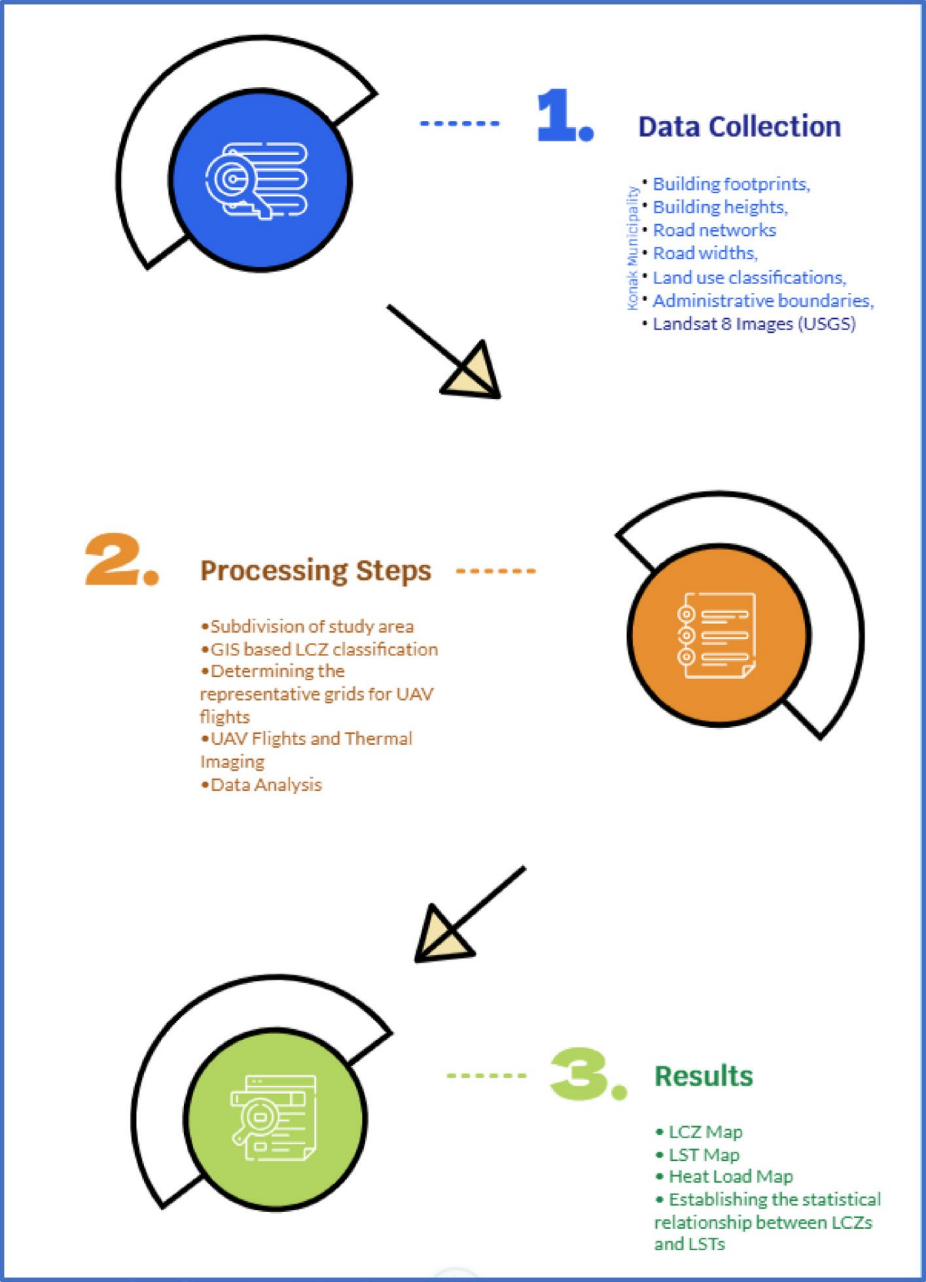
Study workflow.

The study conducted to determine the LCZs for the Konak District and to relate these areas to LST consists of the stages outlined in Fig. 2.

#### Data collection

The initial phase of the research involved gathering spatial and cadastral datasets from the Konak Municipality^33^. These datasets, provided in vector format, included building footprints, building heights, road networks and widths, land use classifications, and administrative boundaries. This information was essential for characterizing the urban environment and understanding the dynamics of the study area. Landsat 8 satellite images were obtained from the USGS^37^ portal. These images were used to derive land use, pervious surface fraction (PSF), and impervious surface fraction (ISF) indicators, all of which contribute to LCZ classification.

#### Subdivision of the study area

LCZs are typically delineated using either city block-based units^38^ or standardized fixed-size grids^39^. A common method involves using 1-ha (100 × 100 m) grids, considered the smallest local spatial unit that significantly influences thermal behavior^39^. Alternatively, some studies determine optimal grid size by analyzing statistical variations in urban form, particularly building height^14,40^. In studies aimed at determining the appropriate grid size for the study area, “spatial autocorrelation,” which arises from the idea that nearby objects tend to show more similarity than distant ones, has been employed. In modeling, semivariance (γ) increases with distance (h) and reaches a maximum level at a threshold distance. In certain situations, when γ no longer changes with distance, this distance is considered the scale of spatial dependence. For instance, Zeng et al.^40^ applied Ordinary Kriging to identify optimal spatial scales in Hong Kong, resulting in a recommended 300 × 300 m grid. However, such approaches require extensive variogram analysis, which was not feasible in this study due to data and temporal constraints. Based on the literature and data characteristics of the study area, a finer-scale 100 × 100 m grid was adopted for all analyses, representing a standard unit in urban thermal studies. The total study area of 2435 hectares has been divided into 2435 grids. Accordingly, the 2435 grids, each covering 1 hectare (100 m × 100 m), together represent the entire 2435-hectare study area.

#### GIS based LCZ classification

Drawing upon spatial datasets from the Konak Municipality and classification criteria proposed in LCZ literature (e.g^6^.,, a set of morphological and surface indicators were selected for determining LCZ types. These analyses, along with the data sources and calculation methods, are presented in Table 1.

**Table 1:** Data and calculation method for local climate zone (LCZ) indicators.

Indicators	Definition	Basic data
BH	Building Height: average building height of each grid, weighted by building floor area (BA: Building area, BAG: Building area of the grid)	Building Data - Basemap $\:BH=\frac{{\sum\:}_{i-1}^{n}{BH}_{i}{BA}_{i}}{{\varSigma\:}_{i-1}^{n}{BAG}_{i}}$
BSF	Building Surface Fraction: Fraction of the total building floor area within the grid (BA: Building area, GA: Grid area	Building Data – Basemap $\:BSF=\frac{{\sum\:}_{i\cdot\:1}^{n}B{A}_{\dot{i}}}{GA}$
AR	Aspect Ratio: Ratio of average building height to average road width	Building ve Road Data- Basemap and OSM
SVF	Skyview Factor: It is the SVF of the study area in an area without buildings (Chen et al., 2010). S_Sky and ∑Sb represent the sky area and the area occupied by buildings at a given point, respectively. SSky+∑Sb represents the entire hemispherical environment at a given point.	$\:SVF=\frac{{\sum\:}_{i\cdot\:1}^{n}SVFi}{n}$ $\:SVFi=\frac{SSky}{(SSky+\:\sum\:\mathrm{S}\mathrm{b}}$
SF	Pervious Surface Fraction: Ratio of pervious areas to grid area	Landsat 8 Satellite Image -USGS
ISF	Impervious Surface Fraction: Ratio of impervious areas to grid area	Landsat 8 Satellite Image -USGS
LU	Land Use: Land use classification was performed via supervised classification of Landsat 8 imagery and validated against municipal cadastral data.	Landsat 8 Satellite Image -USGS

**Fig. 3:**
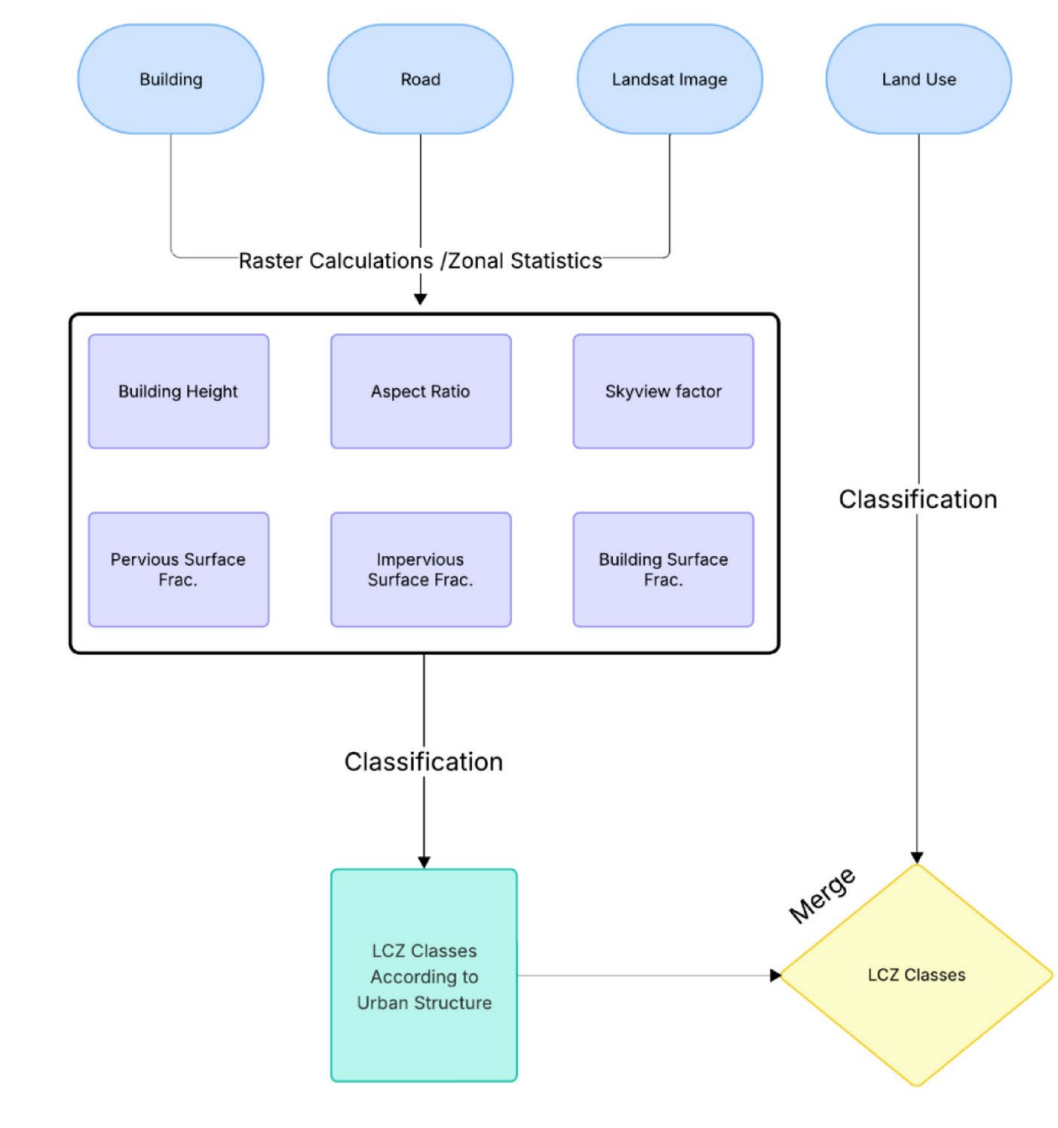
LCZ classification flowchart.

The analyses have been fuzzified according to the ranges obtained in various studies based on the classification by Stewart and Oke^6^. The intervals that emerged as a result of the analyses conducted within the scope of the study and used for LCZ classification are given in the results section. Flowchart of developing LCZ classification is illustrated in Fig. 3.

### Determining the representative grids for UAV flights

The sample size was determined using a standard formula for simple random sampling. There are a total of 2435 squares in the study area. Based on a total population of 2435 units, the minimum required sample size was calculated using the standard formula for finite populations under simple random sampling. At a 95% confidence level and 5% margin of error, the sample size was 332; at 90% confidence, it was 245. However, considering that flights should be conducted with a drone at the same time intervals on days with similar temperatures, it was decided that it would not be feasible to conduct flights over a large number of 100 m x 100 m squares; thus, the total sample size was set at 245 squares with a confidence interval of 90% and a margin of error of 5%.

Following LCZ classification, 14 distinct zone types were identified across the Konak district. Accordingly, the “Stratified Random Sampling” method was chosen to determine the distribution of the total sample size among the LCZ classes. The stratified random sampling method is used in cases where the population is heterogeneous and there is a need to represent different subgroups of the population. Stratified sampling enhances precision in heterogeneous spatial populations by ensuring proportional representation of each stratum, thereby reducing sampling error. This approach is particularly effective in spatial studies where variability within strata is minimized, as demonstrated in recent applications utilizing geospatial technologies^41–43^. For stratified sampling, when the number of cells in each stratum is known, the following formula (1) was used to calculate the sample size for each stratum:

1 $$nk=n\times{Nk/N}$$

*nk *= Sample size for each stratum.

*n* = Total sample size.

*Nk *= Number of cells in each stratum.

*N *= Total number of cells.

To validate the representativeness of the sample across LCZ types, a chi-square goodness-of-fit test was conducted. This test compared the observed frequency of grids within each LCZ (based on the 245-sample distribution) against the expected frequencies calculated from the population LCZ proportions (totaling 2,435 cells). The test statistic was computed as;

$$\:{x}^{2}=\sum\:\frac{{(Oi-Ei)}^{2}}{Ei}$$

where Oi O_i Oi​ is the observed frequency and Ei​ is the expected frequency, with degrees of freedom (df) determined as the number of LCZ categories minus one. This approach ensured that the sampling strategy aligned with the thermal diversity of the study area.

#### UAV flights and thermal imaging

Thermal data were acquired in the selected sample grids using the DJI Matrice 30 T, a commercial-grade UAV equipped with an integrated radiometric thermal camera, selected for its high operational reliability in urban environmental monitoring. The UAV operates within an ambient temperature range of − 20 °C to + 50 °C, with a thermal resolution of 640 × 512 pixels, 40 mm equivalent focal length, 30 fps frame rate, and a measurement accuracy of ± 2 °C under standard atmospheric conditions.

The flights were carried out between May 20 and May 31, at an altitude of 250 m above ground level, during the time period of 11:30 AM to 1:30 PM a time frame selected to minimize diurnal variability in land surface temperature caused by solar angle shifts. Given that the tallest building in the Konak District is approximately 220 m, a flight altitude of 250 m above ground level (AGL) was selected to ensure obstacle clearance. Flights were executed at 7 m/s with image overlap rates of 80% (forward) and 60% (side).

For clarity, UAV flight altitudes may be referenced in three ways: (1) AGL – altitude relative to surface elevation; (2) ATO – fixed altitude relative to takeoff elevation; and (3) AMSL – altitude relative to mean sea level^7^. In this study, AGL was selected to maintain uniform vertical distance across the heterogeneous urban surface.

To address potential temperature fluctuations during UAV operations, all flights were conducted between 11:30 AM and 1:30 PM under clear sky and low-wind conditions. This time window was chosen to minimize the impact of rapid surface temperature variations due to solar angle changes. The DJI Matrice 30 T UAV used in the study features a radiometrically calibrated thermal sensor. A pre-flight stabilization period of 5 min was applied prior to each mission to allow the thermal sensor to reach thermal equilibrium with ambient air temperature, reducing thermal drift and ensuring sensor accuracy^44^. To minimize variability in initial ground surface emissivity and thermal inertia, all UAV missions were launched from asphalt surfaces. These operational controls minimized intra-day LST variability, reducing the need for temporal normalization across the 245 sampled grids and enhancing inter-grid comparability.

#### Data analysis

**Fig. 4:**
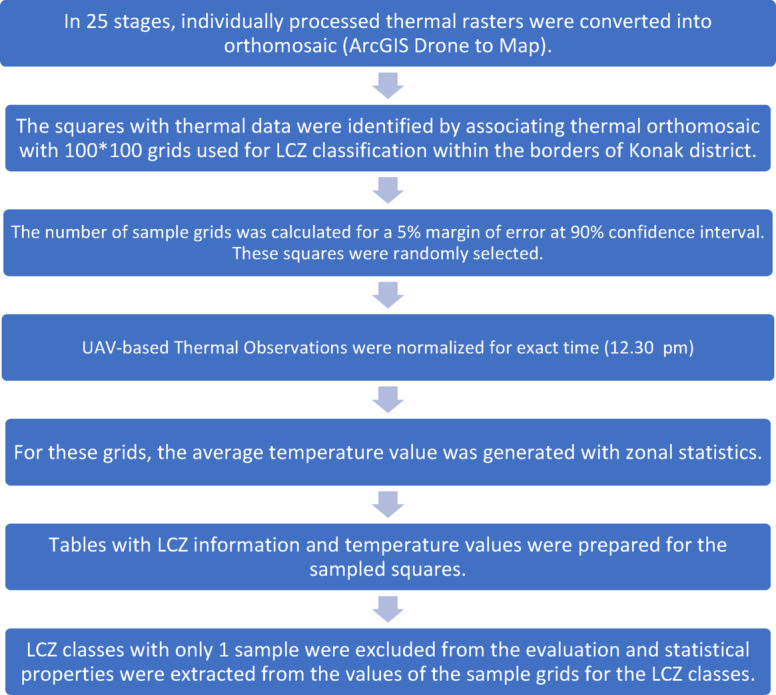
Thermal image processing and spatial analysis workflow for LCZ-based sample grids.

Thermal images collected during UAV flights were processed in ArcGIS Drone2Map to generate radiometrically consistent orthomosaics through image alignment, georeferencing, and mosaicking procedures. Orthomosaics were imported into ArcGIS Pro 3.6, where average land surface temperature values were extracted for each 100 × 100 m grid using the Zonal Statistics tool. The overall thermal data processing workflow, including mosaicking, temperature extraction, and spatial assignment to sample grids, is illustrated in Fig. 4.

#### Temporal normalization of UAV-based thermal observations

To correct for temporal variability in land surface temperature (LST) resulting from UAV flight durations exceeding one hour, a normalization approach was applied using LCZ E (bare rock or paved surfaces) grids, which are known to have low thermal inertia and stable diurnal profiles^6^. Due to their minimal vegetation and high thermal responsiveness, LCZ E areas are particularly suitable for representing short-term diurnal LST fluctuations under stable atmospheric conditions.

Each grid was timestamped according to UAV metadata, and surface temperature was extracted from calibrated thermal imagery with a temporal resolution sufficient to capture within-hour fluctuations. The data were used to construct a temporal LST regression model, forming the basis for interpolating surface temperatures to a common reference time.

All LST values across different LCZ classes were adjusted to a common reference time (12:30 PM) using a linear interpolation based on the LCZ E temperature-time curve. This approach minimized diurnal bias and ensured comparability among grids captured at different times during the UAV survey. The normalization enhances the temporal integrity of the LST dataset, thereby increasing the reliability of spatial analyses comparing thermal profiles across LCZ classes.

#### Establishing the statistical relationship between LCZs and LSTs and creating LST map

After calculating the average LST values for each grid, point representations were generated from the centroids of sampled grids, and spatial interpolation (Inverse Distance Weighting - IDW) was applied to produce a continuous ‘Average Surface Temperature’ map. In addition, a spatial ‘Heat Load’ map was generated to classify the thermal stress levels across Konak District based on standardized LST values.

Heat load can be classified into four categories (optimal, suitable, less suitable, and unsuitable) based on standardized temperature values derived from the applied z-transformation. The formula (2) used for this process is as follows^45^:

2 $$z=(X-\mu)/\sigma$$

where X is the average temperature of a single test cell, µ is the average temperature of all tested cells, and σ is the standard deviation of the average LST of all test cells. Areas with a value less than − 1 in this spatial z-transformation process are classified as having the lowest heat load, while areas with a value greater than 1 are defined as having the highest heat load. A raster was created by applying the formula to all grids with average temperature data, followed by reclassification to produce a map showing the areas classified as optimal, suitable, less suitable, and unsuitable for heat load.

## Results

The results of this study are presented in three subsections to offer a comprehensive and structured analysis of the urban thermal environment in Konak District. Initially, the spatial distribution of Local Climate Zones (LCZs) is examined, followed by a detailed analysis of the thermal characteristics of each LCZ type. Finally, the spatial differentiation patterns of heat load are investigated to highlight the key drivers of thermal stress within the study area.

### Spatial distribution of LCZs in Konak district

**Fig. 5:**
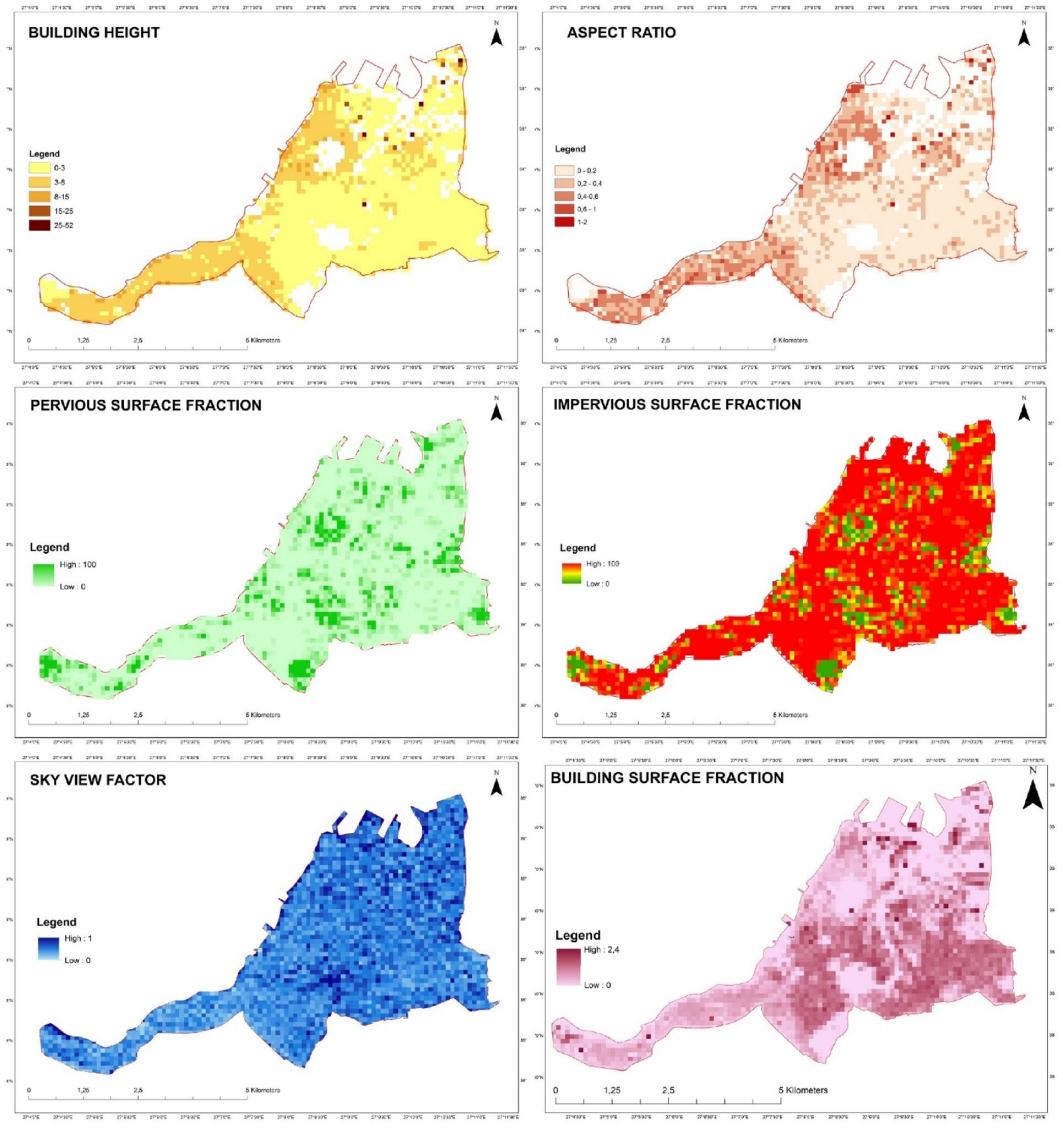
Analysis maps (created by ArcGIS Pro (version 3.6, https://www.esri.com), combining data adapted from^33,37^.

The analysis prepared with the data obtained from Konak Municipality and Landsat 8 satellite images downloaded from the USGS portal are shown in Fig. 5.

These analyses were fuzzified according to the ranges obtained in different studies according to Stewart and Oke^6^ classification. In our study, ranges were determined as shown in Table 2 by examining the building heights, permeable surface area ratio, etc. in the area. Local climate zones marked in red are not included in the study area.

**Table 2:** Geometric, surface cover, and thermal properties of each LCZ type in our study.

	LCZ	BH	BSF	SVF	AR	PSF	ISF
Compact high-rise	1	>=15	40–60	0.2–0.4	> 2	<= 10	40–60
Compact midrise	2	5–15	40–70	0.3–0.6	0.75–2.75	< 20	50–100
Compact low-rise	3	1–5	40–70	0.2–0.6	0.75–1.5	=< 30	50–100
Open high-rise	4	<=15	20–40	0.5–0.7	0.75–1.25	30–40	30–50
Open midrise	5	5–15	20–40	0.5–0.8	0.3–0.75	20–40	30–50
Open low-rise	6	1–5	20–40	0.6–0.9	0.3–0.75	30–60	20–50
Lightweight low-rise	7	1–3	60–90	0.2–0.5	1–2	< 30	< 20
Large low-rise	8	1–5	30–50	> 0.7	0.1–0.3	=< 20	40–100
Sparsely built	9	1–10	10–20	> 0.8	0.1–0.25	60–80	< 20
Heavy industry	10	2–10	20–30	0.6–0.9	0.2–0.5	40–50	20–40
Dense trees	A	0	< 10	< 0.4	> 1	> 90	=< 10
Scattered trees	B	0	<= 10	0.5–0.8	0.25–0.75	> 90	<= 10
Bush. scrub	C	0	=< 10	0.7–0.9	0.25–1.0.25.0	> 90	<= 10
Low plants	D	0	< 10	> 0.9	< 0.1	> 90	< 10
Bare rock or paved	E	0	< 10	> 0.9	< 0.1	=< 10	> 90
Bare soil or sand	F	0	=< 10	> 0.9	< 0.1	> 90	<= 10
Water	G	0	< 10	> 0.9	< 0.1	> 90	< 10

**Fig. 6:**
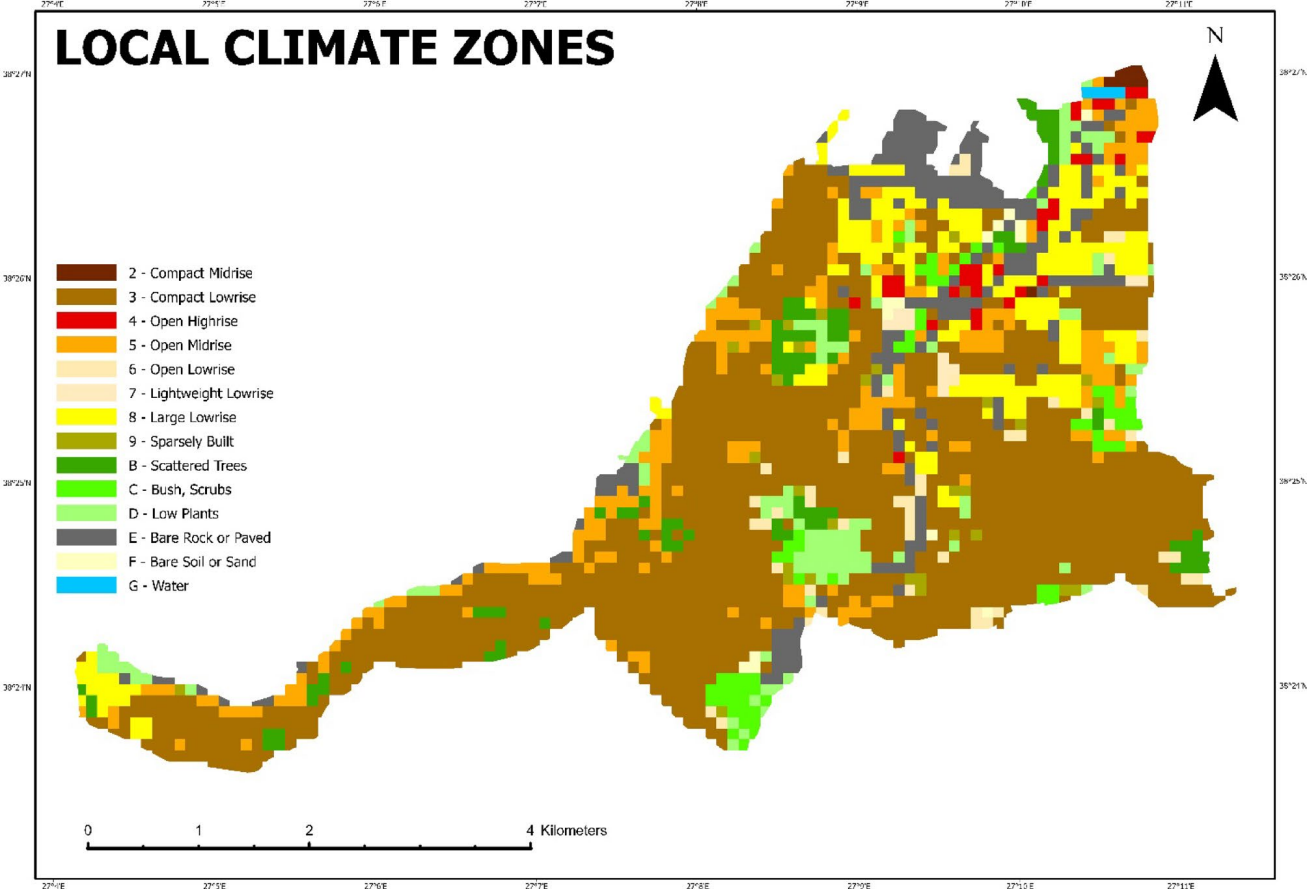
Local climate zones of Konak district (Prepared by the author using ArcGIS Pro (version 3.6, https://www.esri.com)).

As a result of these analyzes, the local climate zones map of Konak district, created as described in the methodology section, is presented in Fig. 6.

In the study, it was calculated that 245 grids were sufficient with a 90% confidence interval and 5% margin of error. This ensured that the selected sample was statistically robust enough to represent the variability across LCZ types while maintaining a manageable number of UAV flights. The distribution of these squares according to the LCZ classes is presented in Table 3, demonstrating balanced coverage across different urban morphologies.

**Table 3:** Distrubition of LCZs.

LCZ	Number of cells	Surface (ha)	% of study area	Simple random sampling - %90 confidence interval and %5 error margin	
				Calculated sample size	Rounded sample size
2	8	7.2	0.30	0.7	1
3	1340	1321.5	55.33	135.5	136
4	34	33.25	1.39	3.4	3
5	242	239.5	10.03	24.56	25
6	48	47	1.97	4.8	5
7	12	12	0.50	1.23	1
8	221	219	9.17	22.46	22
9	31	31	1.30	3.17	3
B	96	93.2	3.90	9.56	10
C	65	63	2.64	6.46	6
D	112	104.7	4.38	10.74	11
E	208	199	8.33	20.41	20
F	14	14	0.59	1.43	1
G	4	4	0.17	0.41	1
TOTAL	2435	2388.4	100.00	244.83	245

### Analysis of thermal environment characteristics of different LCZ types

The 245 grids, selected using stratified random sampling proportional to the LCZ area distribution within the 2,435-cell grid system of Konak district. A chi-square test validated the representativeness, yielding a p-value of 0.99 (df = 13, *p* > 0.05), indicating that the sample distribution aligns with the population LCZ proportions. This test is confirming that the sample adequately represents the dominant LCZ types (3, 5, 8, B, D, and E), which collectively account for approximately 92% of the total classified area. Single-sample LCZ classes (e.g., LCZ 2 and 7) were excluded to ensure statistical robustness, as their limited representation could skew variance estimates, a decision supported by the methodology’s focus on reliable thermal pattern analysis. This sampling strategy ensures a representative coverage of thermal conditions across the study area’s urban typologies, as evidenced by the consistent temperature rankings (Spearman’s ρ ≈ 0.99).

**Fig. 7:**
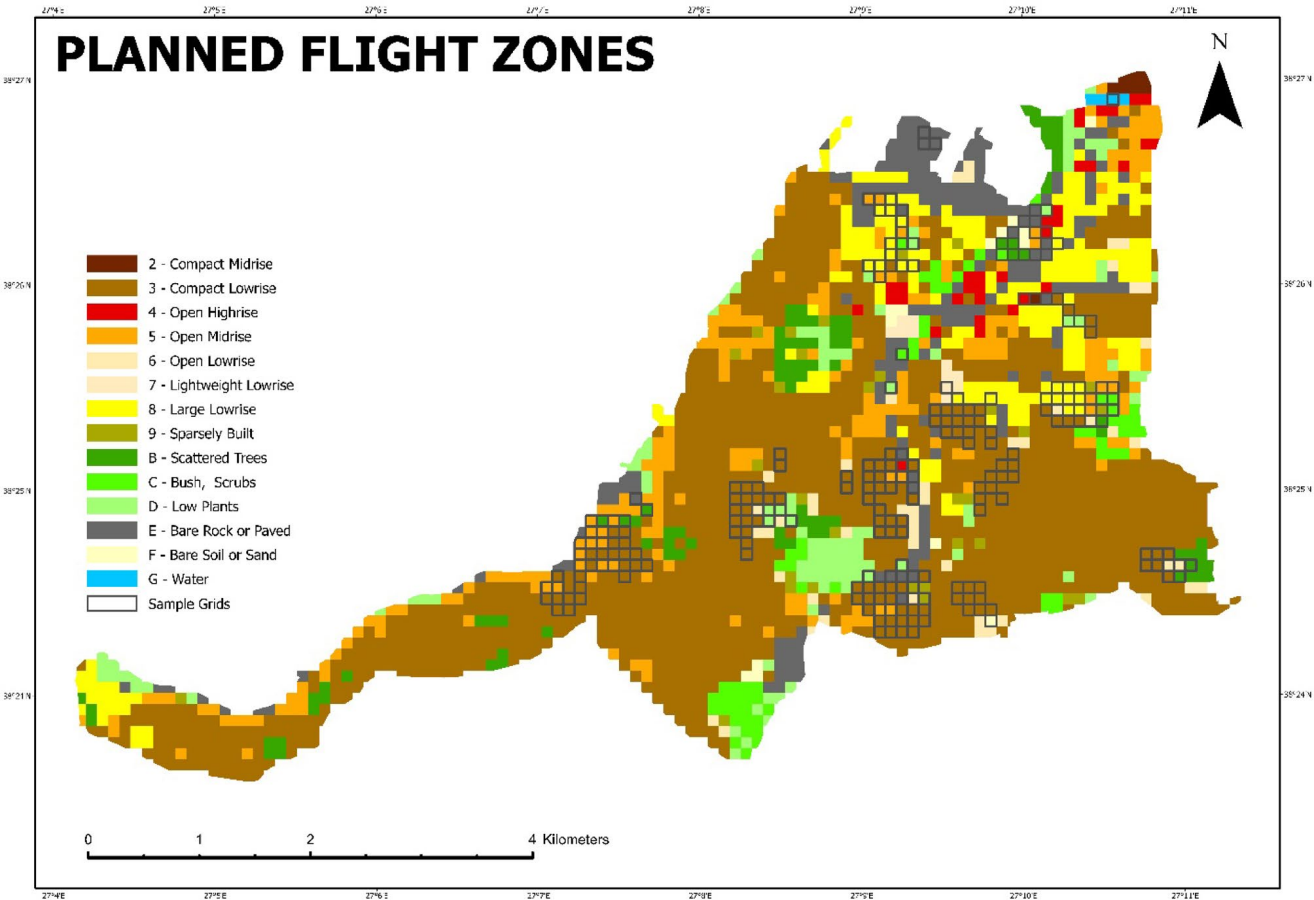
Planned flight zones for simple random sampling (%90 Confidence Interval and %5 Error Margin) (Prepared by the author using ArcGIS Pro (version 3.6, https://www.esri.com).

After calculating the sample size for each LCZ, the selection of squares for flying was determined by opening the attribute table in GIS software, and a random selection was made for each LCZ class. This randomization reduced potential selection bias and guaranteed that both densely built and vegetated LCZs were proportionally included in the analysis. The squares planned for flights are shown in Fig. 7, forming the foundation for subsequent thermal comparisons between LCZ classes.

**Fig. 8:**
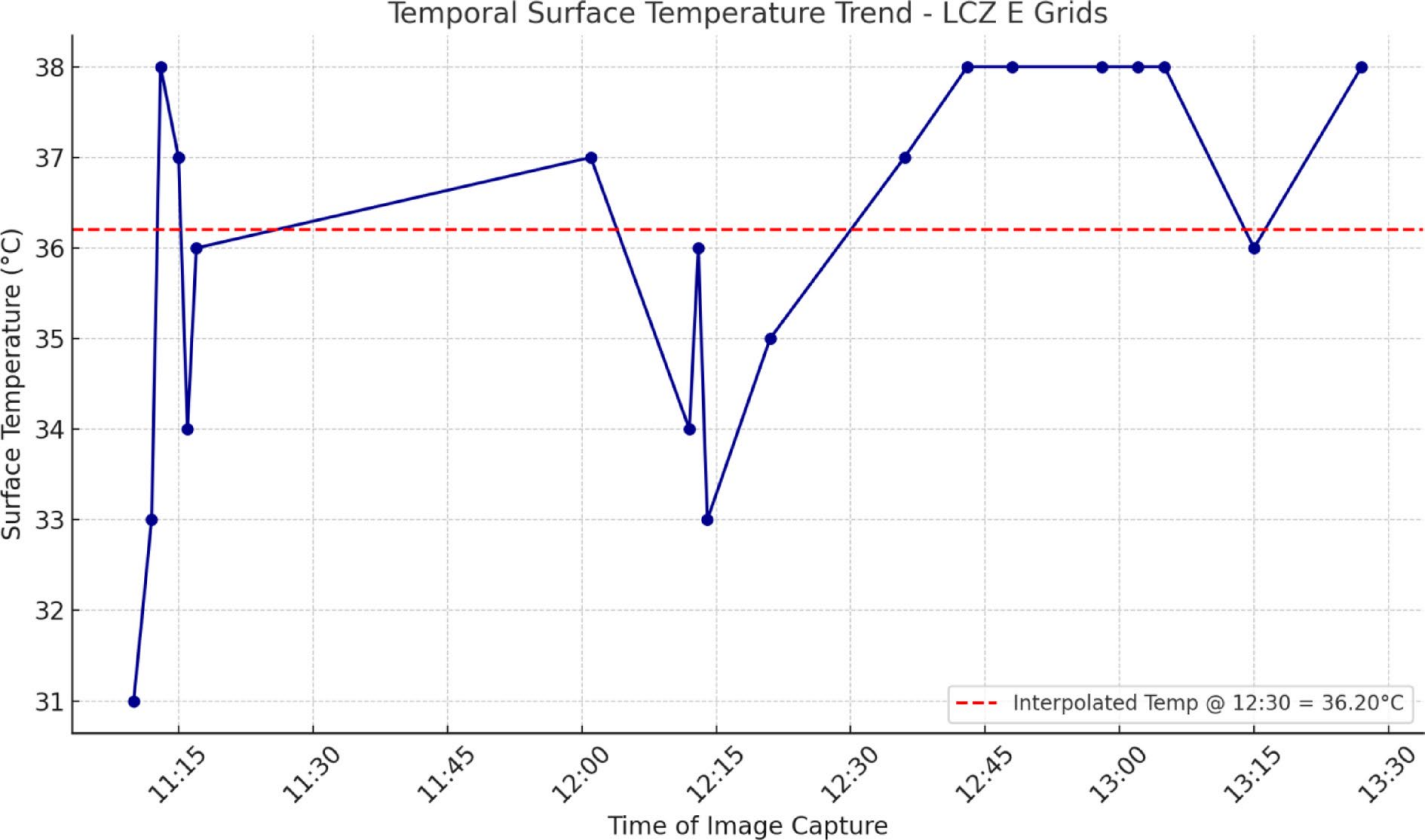
Temporal surface temperature trend for LCZ E grids.

Although the UAV flights for surface temperature measurement was conducted during the peak midday hours, specifically between 11:30 AM and 1:30 PM, temporal normalization was deemed necessary to account for the intra-flight temperature variations. As described in the methodology section, this correction was based on 20 grids classified as LCZ E, which represent paved or rocky surfaces known for their thermal responsiveness. A temperature-time trend was derived from these LCZ E grids (Fig. 8), and all surface temperature values were adjusted to a reference time of 12:30 PM accordingly.

Following the assignment of temperature values to each sample grid via UAV-based orthomosaics, statistical analyses were performed to evaluate the temperature characteristics of each LCZ class. In addition, normalized LST values—adjusted to 12:30 PM using the LCZ E trend—were also calculated for all LCZ types as shown in Formula (3). These temporally corrected values are presented in Table 4.

3 $$\text{Normalized Mean LST = Mean LST + (Reference LCZ E Temperature at 12:30 - Mean Temperature of LCZ E)}$$

**Table 4:** Statistical properties calculated from example LCZ grids.

LCZ_CLASS	Frequency	Min.temp.	Max.temp.	Mean temp.	Standard deviation	Coefficient of variance	Median temp.	Normalized mean temp. (12:30)
2	1	33	33	33.00	0.00	0.00	33.00	33.45
3	136	22	42	35.95	3.48	12.12	36.00	36.40
4	3	34	34	34.00	0.00	0.00	34.00	34.45
5	25	30	35	32.28	1.54	2.38	33.00	32.73
6	5	30	36	33.20	2.59	6.70	34.00	33.65
7	1	41	41	41.00	0.00	0.00	41.00	41.45
8	22	27	38	32.05	3.11	9.66	32.00	32.50
9	3	29	33	30.67	2.08	4.33	30.00	31.12
B	10	29	33	31.00	1.56	2.44	31.00	31.45
C	6	28	41	31.67	4.72	22.27	30.50	32.12
D	11	26	43	33.18	5.44	29.56	32.00	33.63
E	20	28	38	35.75	2.61	6.83	37.00	36.20
F	1	33	33	33.00	0.00	0.00	33.00	33.45
G	1	23	23	23.00	0.00	0.00	23.00	23.45

Since LCZ 2, LCZ 7, LCZ F and LCZ G are represented by only one grid, these values are accepted without statistical processing for the grids belonging to these classes.

Accordingly, LCZ 7 (Lightweight Lowrise), LCZ 8 (Large Lowrise) and LCZ E (Bare Rock or Paved) have the highest mean and median temperature values. In these areas, metal roofing and asphalt surfaces raise the temperature considerably. In addition, the lowest mean and median temperature values were observed in LCZ G (Water), followed by LCZ B (Scattered Trees). The cooling effect of water and vegetation was once again observed.

**Fig. 9:**
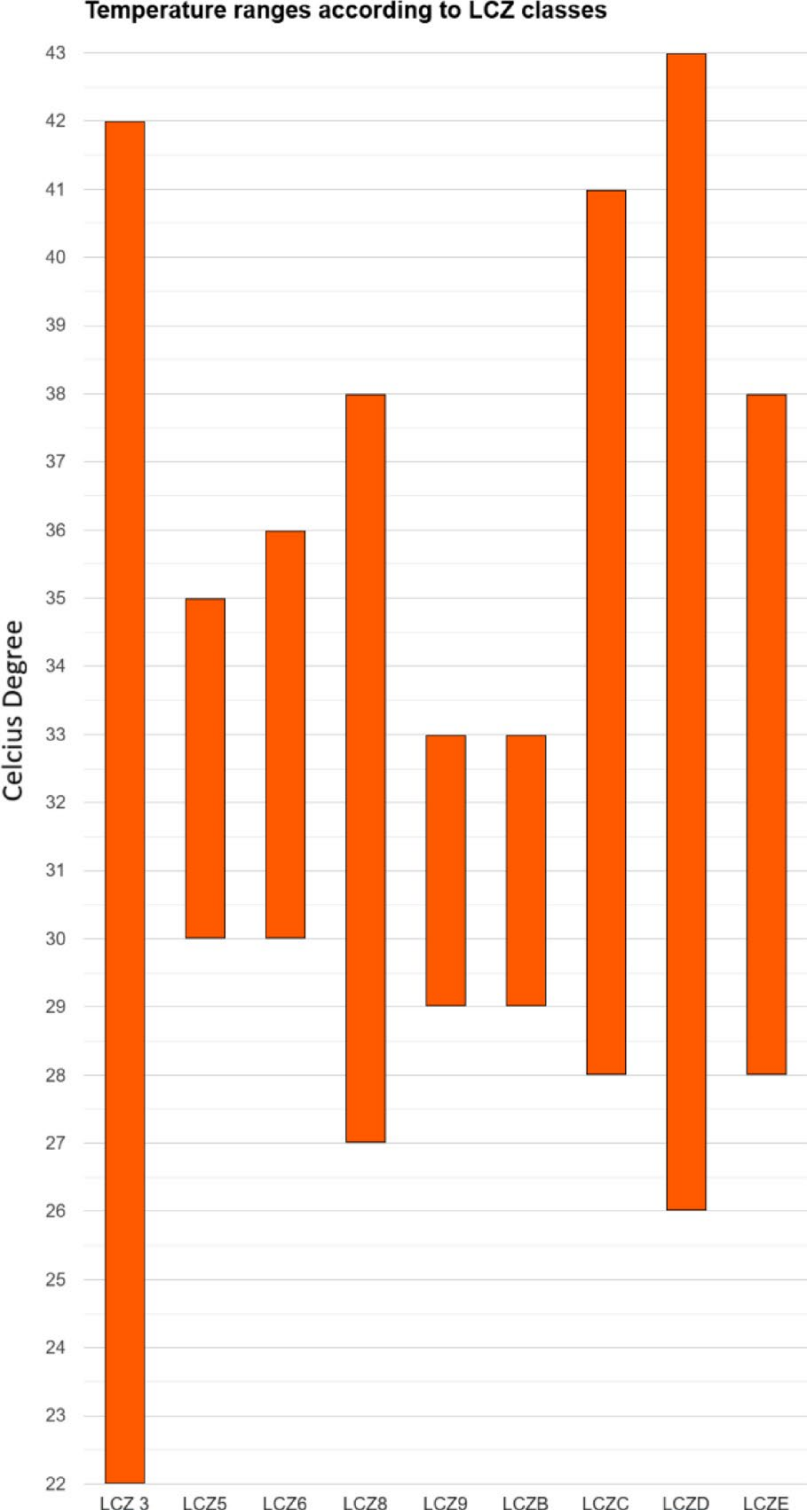
LCZ Classes’ Box-Plot Graph.

For a better understanding of the temperature ranges of LCZ classes, box-plot graphs were prepared by excluding the classes with no difference between minimum and maximum values. As seen in Fig. 9, the largest temperature ranges were observed in LCZ 3 (20 °C), LCZ D (17 °C), LCZ C (13 °C) and LCZ 8 (11 °C) classes.

### Spatial differentiation patterns of heat load

After obtaining the average temperature value for each LCZ class, a raster was created by entering the average temperature data in all grids. Using this raster, the “Heat Load” map, which shows the spatial distribution of different degrees of heat load, was prepared by raster calculation using the formula detailed in the method section.

**Fig. 10:**
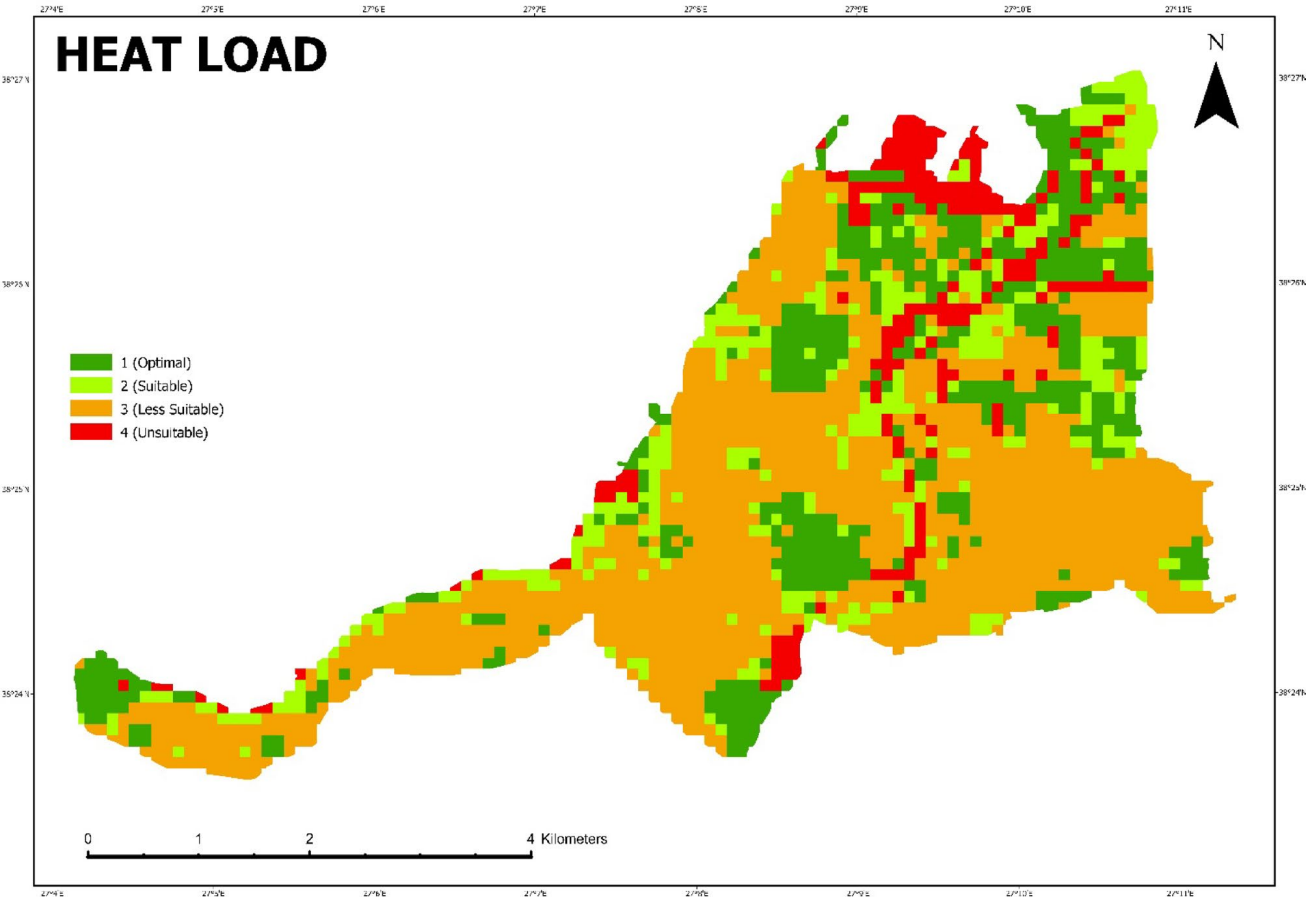
Heat Load Map (Prepared by the author using ArcGIS Pro (version 3.6, https://www.esri.com).

As a result of this process, which is also defined as the spatial z-transformation process, areas with a value less than − 1 are defined as areas with the lowest heat load and areas with a value greater than 1 are defined as areas with the highest heat load (Fig. 10).

LCZ classes’ mean temperature: 35.16 °C.

LCZ classes’ standart deviation: 2.12 °C.

As can be seen in this map, the parts of the city with high-rise buildings, wide roads and concrete and asphalt surfaces such as the harbor are the areas with the highest heat load and cover 8.8% of the city, as can be seen in Table 5. Open green areas and wooded areas have the lowest heat load and cover 21.7% of the city’s surface area.

**Table 5:** Heat load distrubition.

Heat load categories	z-Scores	Area %
1 (Optimal)	<−1.00	21.7
2 (Suitable)	from − 0.99 to 0.00	14.5
3 (Less Suitable)	from 0.01 to 0.99	55
4 (Unsuitable)	> 1.00	8.8

**Fig. 11:**
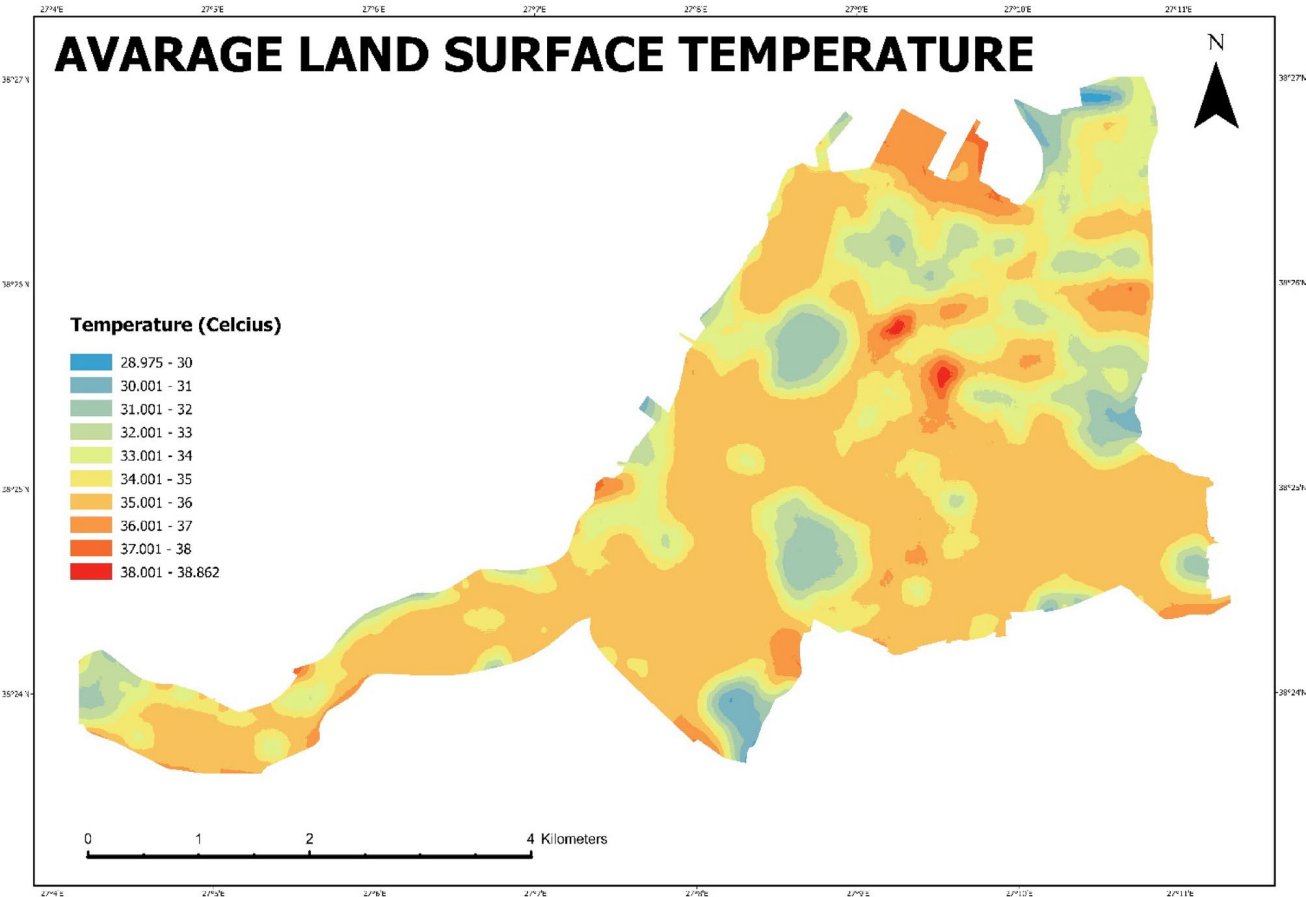
Avarage land surface temperature (Prepared by the author using ArcGIS Pro (version 3.6, https://www.esri.com).

A geostatistical analysis is required for the spatial distribution of surface temperature. For this purpose, a polynomial local interpolation approach used to create surfaces based on the locations of predicted values and measured values. The result of the spatial interpolation clearly shows that there are poly-structural (polycentric) urban heat islands in Konak District, but these centers are mostly clustered in the north (Fig. 11).

## Evaluation and discussion

### Contributions and methodological advancements

This study advances urban climatology by integrating high-resolution UAV-derived thermal imagery with GIS-based Local Climate Zone (LCZ) classification to assess intra-urban temperature dynamics in Konak, İzmir. The combination of a 100 m × 100 m grid system and stratified random sampling ensured spatially representative thermal assessments across 14 LCZ types. Our findings indicate that zones with high impervious surface fractions—such as LCZ 7 (Lightweight Low-rise), LCZ 8 (Large Low-rise), and LCZ E (Bare Rock or Paved)—consistently exhibited elevated mean land surface temperatures (LSTs), while vegetated and water-rich zones (e.g., LCZ B and LCZ G) showed noticeable cooling effects. These results are in alignment with existing literature^6^ and underscore the role of land cover in shaping urban microclimates.

Unlike satellite-based analyses limited by lower spatial resolution and temporal frequency, the UAV-based method employed here captures fine-scale temperature variations at the neighborhood level. The implementation of a temporal normalization procedure using LCZ E reference grids allowed for adjustment of intra-flight thermal fluctuations, enhancing the comparability of LST data across grids. This refinement is rarely used in UAV-based urban climate studies and represents a methodological contribution that improves data integrity and spatial consistency.

The combination of high-resolution UAV thermal imaging and Local Climate Zone (LCZ) analysis offers several advantages over traditional satellite remote sensing methods for urban planning. UAV thermal imaging provides much finer spatial resolution, allowing for detailed temperature mapping of individual buildings, streets, and small urban features that may be missed by coarser satellite data. This granular view enables planners to identify localized hot spots and cool areas within the urban fabric with greater precision. When integrated with LCZ classification, which characterizes urban morphology and land cover types, planners can better understand how specific urban forms and materials influence the thermal environment at the neighborhood scale.

### Comparative analysis and contextual interpretation

When compared with studies from other urban contexts, the observed thermal patterns in Konak reveal both consistent and context-specific behaviors. For example, elevated temperatures in LCZ 8 are consistent with findings from Bangkok and Hong Kong^40,46^, where extensive asphalt and metal surfaces contribute to heat retention. Meanwhile, lower LSTs in LCZ B and G are comparable to cooling trends observed in Lisbon^47^. However, the wider LST range observed in Konak’s LCZ 3 (Compact Low-rise) compared to Athens^15^ may reflect Konak’s complex urban morphology, including dense commercial areas like the Kemeraltı Bazaar and limited urban shading.

Such differences also highlight the significance of local building materials, surface characteristics, and ventilation conditions. For instance, the higher LSTs in Konak’s LCZ E compared to Hong Kong may be attributed to the prevalence of asphalt surfaces, which have higher thermal emissivity than concrete. Furthermore, unlike the monocentric UHI pattern reported in cities like Sofia^25^, Konak exhibited a polycentric UHI structure, with multiple heat accumulation zones identified in the northern harbor and commercial districts.

For high-density Mediterranean cities like İzmir, these findings have particular value in addressing urban heat challenges. The detailed thermal and LCZ data can guide targeted interventions to mitigate urban heat island effects, such as identifying optimal locations for green spaces, recommending building materials and designs that reduce heat absorption, and planning street orientations that maximize natural ventilation. Planners can use this information to develop climate-responsive urban designs that improve thermal comfort for residents while reducing energy demands for cooling. Additionally, the high-resolution data enables more accurate modeling of future climate scenarios at the city scale, allowing planners to anticipate and prepare for long-term temperature changes in the Mediterranean urban context.

### Practical implications for urban planning

The heat load map generated from normalized LST data shows that approximately 8.8% of Konak’s urban area experiences high thermal stress, primarily concentrated in dense, impervious zones, while 21.7% benefits from optimal thermal conditions due to green and blue infrastructure. These results provide actionable insights for climate-responsive planning. To make the Konak District and İzmir “cooler,” our findings have direct, actionable guiding value. The analysis identified specific LCZ types with the highest heat load, such as Compact Low-Rise (LCZ 3) and Compact Mid-Rise (LCZ 2), which are the primary targets for urban heat mitigation. Based on these findings, we propose a multi-faceted approach centered on proactive retrofitting and urban design strategies.

First, prioritizing green infrastructure is essential. Our results highlight the significant cooling effect of vegetated zones, emphasizing the need to integrate urban forestry, pocket parks, and green roofs and walls into high-heat-load areas^48^. These measures enhance evapotranspiration and provide shade, effectively reducing both surface and air temperatures.

Second, the modification of urban materials is a critical strategy. In areas with extensive impervious surfaces, we recommend replacing dark, absorptive materials with those of high-albedo. This includes using light-colored pavements, cool roofs, and reflective coatings on buildings to increase solar reflectance and reduce heat absorption.

Finally, optimizing urban form is key to promoting passive cooling. We recommend adjusting building orientations to maximize natural airflow and creating urban ventilation corridors. The implementation of water features, such as fountains and misting systems in public spaces, can also create localized cooling microclimates. Furthermore, encouraging the use of shading devices on buildings and in public areas can reduce direct solar radiation and enhance pedestrian thermal comfort^49^.

These recommendations should be integrated into a comprehensive urban heat island mitigation plan that aligns with zoning regulations and building codes. Engaging local communities in the planning process and educating residents about heat-reducing practices will be crucial for the success of these interventions. Regular monitoring and assessment of temperature changes across different LCZs will help evaluate the effectiveness of these strategies and guide future planning decisions. This study thus provides a tangible roadmap for planners to transition from broad, city-wide strategies to precise, neighborhood-level interventions, directly contributing to the “Cooler Cities” theme and enhancing urban resilience in Mediterranean climates.

### Limitations and recommendations for future work

Several limitations of this study should be acknowledged. First, the thermal data were collected during a limited time window (late May, mid-day hours), which may not fully capture seasonal or diurnal variations in surface temperature. Atmospheric factors such as wind, humidity, and partial cloud cover during UAV flights may also introduce variability in the recorded temperatures, highlighting the sensitivity of UAV-based measurements to weather conditions. Second, while the sample of 245 grids ensured LCZ representation at a 90% confidence level, additional sampling across different seasons and times of day could strengthen the robustness of the thermal profiles.

The accuracy specification of the thermal camera used in this study is ± 2 °C. While this introduces a degree of uncertainty in absolute temperature values, the observed inter-class differences between LCZs (typically ranging from 4 to 8 °C) exceed this margin, supporting the robustness of the relative thermal patterns identified. Similar levels of sensor accuracy have been reported as acceptable in UAV-based LST studies (e.g^17,50^.,. Nevertheless, this margin should be considered when interpreting the results, particularly for LCZ classes with relatively small thermal contrasts.

Moreover, although İzmir shares key climatic and morphological characteristics with other Mediterranean cities, its unique urban morphology (e.g., compact commercial cores, industrial waterfronts) constrains the direct generalization of the results. Nevertheless, the methodological framework presented here—combining UAV-based LST measurements with LCZ classification—is transferable and adaptable to other Mediterranean urban contexts.

This study also did not integrate socioeconomic or demographic variables, which are essential for understanding the intersection of thermal exposure and urban vulnerability. Including such data would enhance the capacity of the model to support equitable climate adaptation strategies.

Future research should expand UAV flights to capture seasonal and hourly variations in LST, allowing for a more comprehensive understanding of temporal dynamics. Integrating social vulnerability indicators into spatial thermal analyses would also enhance the capacity to link thermal exposure with urban resilience and equity considerations. In addition, testing the methodology in other Mediterranean cities could provide insights into the generalizability of the LCZ–temperature relationship beyond the case of İzmir. Finally, simulating future climate scenarios and evaluating adaptation interventions—such as reflective surfaces, cool roofs, or urban greening—would strengthen the practical relevance of the approach for climate-sensitive urban planning^51,52^.

## Conclusion

The findings of this study provide actionable insights for urban planning and climate adaptation. In zones with elevated heat loads such as LCZ 7 (Lightweight Low-rise) and LCZ 8 (Large Low-rise), targeted interventions could include the introduction of reflective roofing materials, permeable pavements, and increased tree canopy cover to mitigate excessive surface heating. Or increasing green coverage in LCZ 3 (compact low-rise) areas by 15–20% to reduce LST by up to 2–3 °C, as observed in the study’s heat load data. In contrast, cooling zones such as LCZ B (Scattered Trees) and LCZ G (Water) highlight the critical importance of preserving and expanding green and blue infrastructure within urban planning strategies. The heat load maps produced here can serve as decision-support tools for municipalities, helping to prioritize investment in vulnerable neighborhoods, design heat-resilient public spaces, and integrate climate-sensitive zoning regulations. By linking LCZ-based thermal analyses to planning actions, this methodology strengthens the policy relevance of UAV and GIS-based urban climate research. These strategies are adaptable to other Mediterranean cities with similar LCZ distributions, such as Athens or Valencia, pending local climate adjustments.

By addressing the spatial heterogeneity of urban heat patterns through a high-resolution, UAV-GIS integrated approach, this study provides a replicable and actionable framework for urban climate analysis. However, to fully inform climate-resilient and socially inclusive planning, future work must go beyond physical variables to incorporate social dimensions and broader temporal coverage.

In summary, this study demonstrates that UAV–LCZ integration can reliably capture fine-scale thermal differences across urban morphologies, highlighting priority areas for intervention (e.g., LCZ 7, LCZ 8, and LCZ 3). By combining detailed temperature mapping with actionable planning recommendations, it bridges the gap between scientific analysis and policy application, while also setting the stage for future research that expands across seasons, integrates social vulnerability, and tests adaptability in other Mediterranean cities.

## Data Availability

The datasets generated and/or analyzed during the current study are not publicly available due to institutional restrictions imposed by Konak Municipality, as the thermal UAV imagery and related geospatial data are proprietary to the municipality. However, these datasets are available from the corresponding author on reasonable request, subject to permission from Konak Municipality.
